# Unwanted Diagnosis of a Subcutaneous Swelling

**DOI:** 10.7759/cureus.8141

**Published:** 2020-05-15

**Authors:** Indira Ramasamy, Ashok Kumar Sahoo, Harish Goutham Medapati, TP Elamurugan, Vishnu Prasad Nelamangala Ramakrishnaiah

**Affiliations:** 1 Surgery, Jawaharlal Institute of Postgraduate Medical Education & Research, Puducherry, IND; 2 Surgical Gastroenterology, Government Medical College, Srinagar, IND; 3 Surgery, Jawaharlal Institute of Postgraduate Medical Education and Research, Puducherry, IND

**Keywords:** pseudohyphae, epidermal inclusion cyst, melanin, immunocompromised, histopathology

## Abstract

Subcutaneous swelling is one of the common cases seen in surgical practice. The pathology of the subcutaneous swellings is varied ranging from epidermal inclusion cyst to malignant swelling. Fungal infections producing subcutaneous swelling are relatively rare. They occur in immunocompromised patients. We report a case of phaeohyphomycosis (PHM) which is characterized by the presence of pseudohyphae, hyphae, brown yeast-like cells, and melanin in their cell walls, presenting as subcutaneous swelling.

A 34-year-old male presented with a swelling over the anterior aspect of left knee joint for three months, which was initially painless. He gave a history of purulent discharge from the swelling 20 days back. He was a known case of myasthenia gravis on regular treatment with steroids. On examination, the swelling was firm, nontender, and mobile in subcutaneous plane. The skin over the swelling showed a healed puckered scar, fine needle aspiration cytology (FNAC) of the swelling showed slender, septate hyphae with variable branching bulbous ends, and few of the hyphae showed pigmentation morphologically suggestive of PHM. The swelling was excised with clear margin.

Subcutaneous mycosis is common in tropical and subtropical countries like India. Strong suspicion of this diagnosis is warranted especially in immunocompromised patients. Surgical excision is the treatment of choice to achieve early cure.

## Introduction

Any fungus in tissue with pseudohyphae, hyphae, brown yeast-like cells, or a combination of these forms is well described under term phaeohyphomycosis (PHM) [1]. It is a relatively new term introduced by Ajello in 1974 [2]. These organisms are characterized by the presence of melanin in their cell walls, which is a prime virulent factor in these patients [3]. At least 60 genera and 109 species of fungi are found to cause PHM. Exophiala spinifera, Wangiella spinifera, Phialophora spinifera, and Bipolaris spinifera are most frequently encountered etiologic agents [4]. Although the fungi causing PHM are found across the world, they are more commonly encountered in tropical and subtropical areas [5]. PHM is found to occur in both immunocompromised and immunocompetent persons, but more frequent in immunocompromised patients. Post organ transplantation, cancer, leukemia, prolonged hospitalization, and corticosteroid therapy are predisposing factors [6,7]. PHM can be classified based on the site of localization into superficial, cutaneous and corneal, subcutaneous, and systemic [6]. This present case falls in the subtype of subcutaneous PHM in a patient on corticosteroids.

## Case presentation

**Figure 1:**
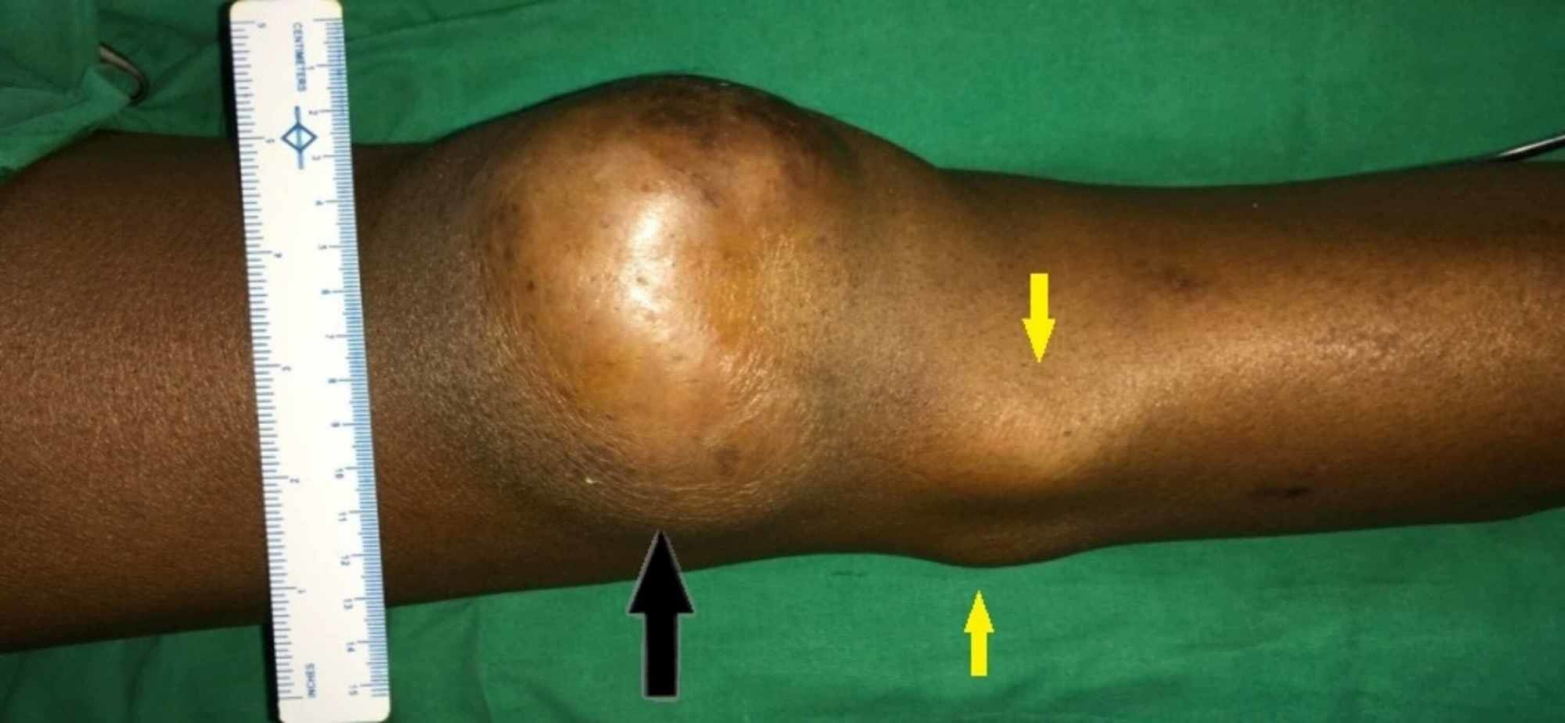
A bigger swelling on the right knee joint (black arrow) and other small swellings (yellow arrows) in the subcutaneous plane in the right leg

A 34-year-old male presented with complaints of swelling on the anterior aspect of the left knee joint for three months. It was insidious in onset and slowly progressively increasing in size. The swelling has been associated with moderate dull aching pain for 10 days. Swelling had ruptured 20 days before presentation with pus discharge, but there was no decrease in the size of the swelling. He gave no history of fever or any complaints in movements at the knee joint. The patient was diagnosed as a case of myasthenia gravis, and he has been on steroids since then. The patient had also developed steroid-induced diabetes mellitus. The patient was admitted with complaints of weakness of bilateral upper and lower limbs. On examination, there was a swelling on the right knee joint about 15 × 10 cm in size in the subcutaneous plane, and also three more swellings of maximum size 4 × 5 cm in the subcutaneous plane in the anterolateral aspect of the upper two-third of the right leg (Figure 1).

**Figure 2:**
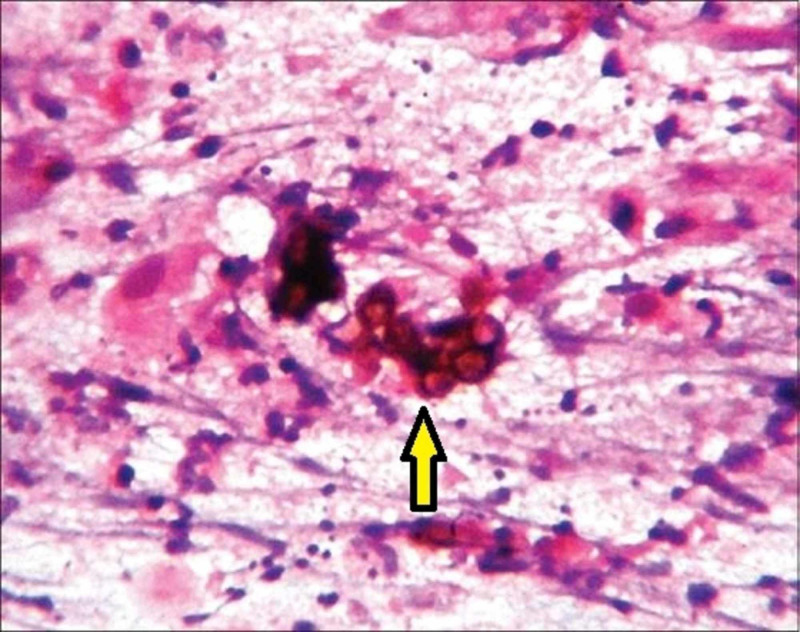
Fine needle aspiration cytology of the swelling showing fungal organisms with few of the hyphae having pigmentation morphologically suggestive of phaeohyphomycosis (yellow arrow)

The patient was evaluated. Fine needle aspiration cytology (FNAC) of the swelling showed sheets of neutrophils in a necrotic background. There were many fungal organisms, which are slender, septate hyphae with variable branching bulbous ends. A few of the hyphae had pigmentation morphologically suggestive of PHM (Figure 2).

**Figure 3:**
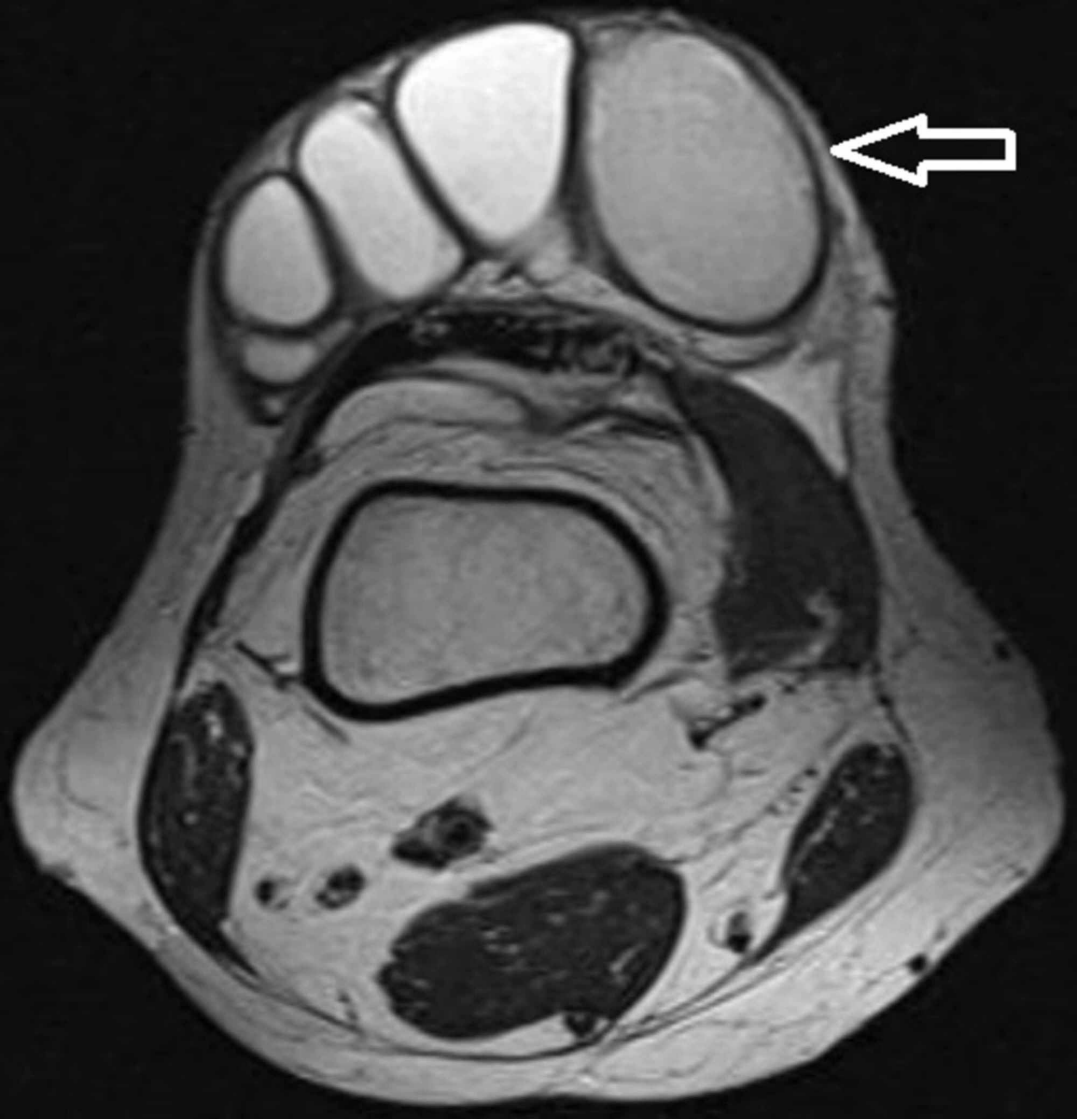
MRI of the right knee (transverse plane) showing hypoechoic cystic loculated lesion in subcutaneous plane (arrow)

**Figure 4:**
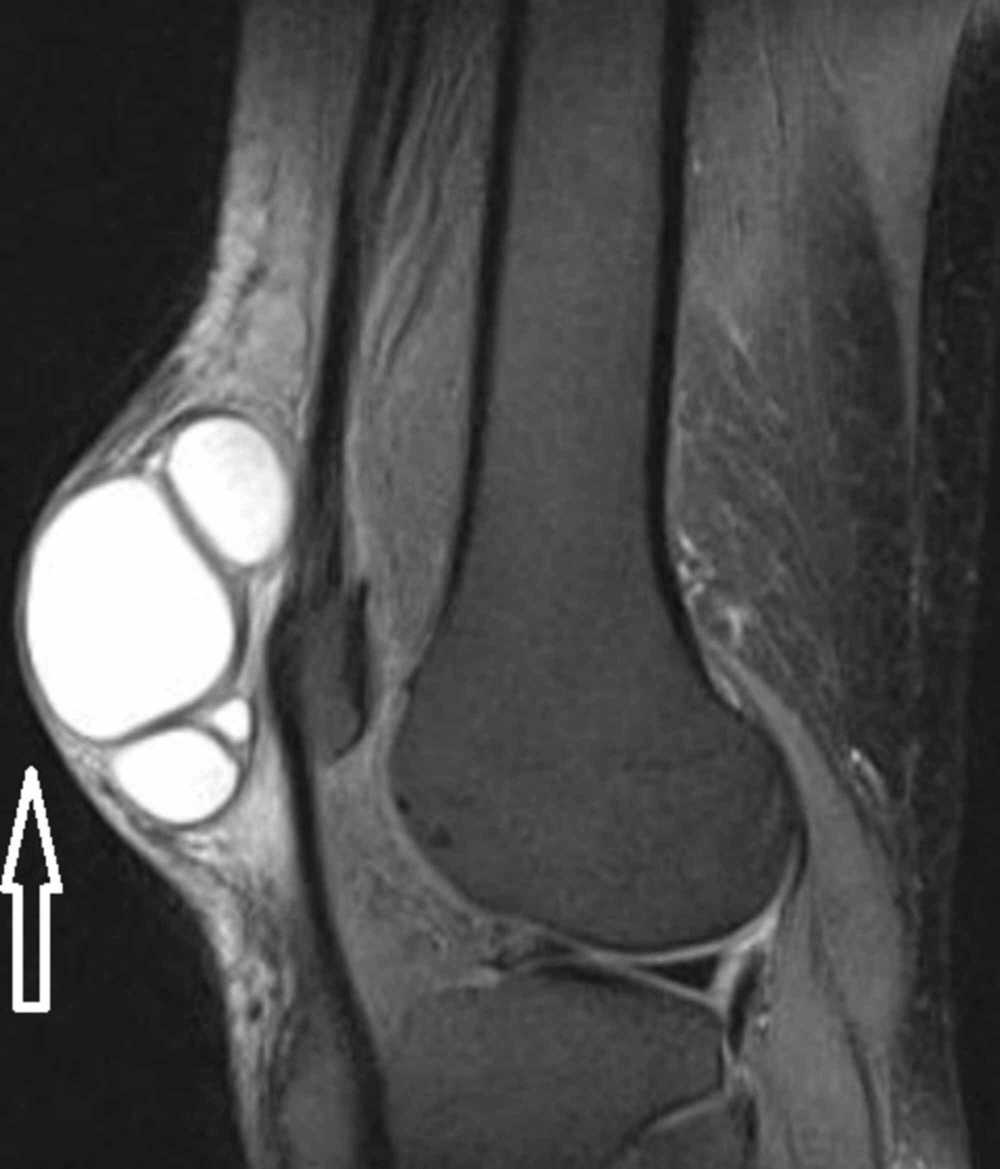
MRI of the right knee (coronal view) showing hypoechoic cystic loculated lesion in subcutaneous plane (arrow)

Periodic acid-Schiff (PAS) stain had highlighted the fungal organism. MRI of the right lower limb was done, which showed a hypoechoic cystic loculated lesion in the subcutaneous plane with no involvement of the underlying knee joint (Figures 3, 4).

**Figure 5:**
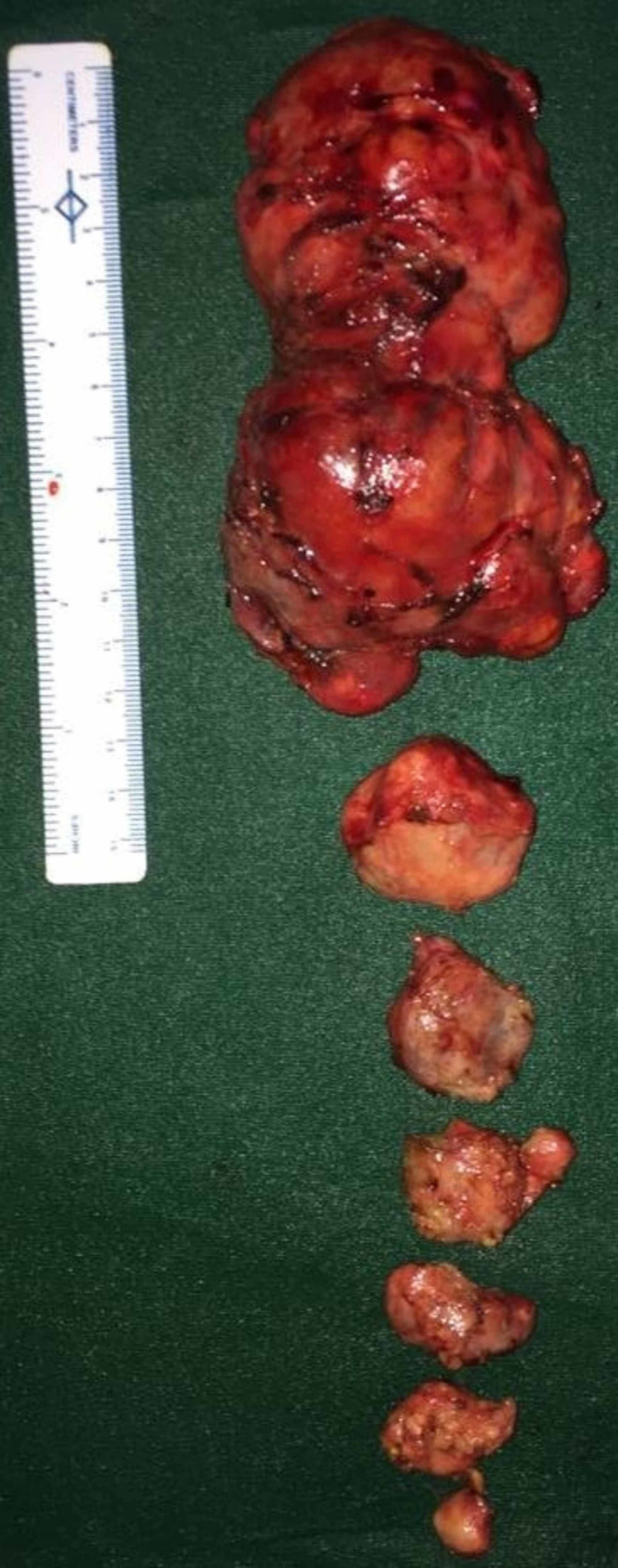
Excised specimen

The patient was optimized for surgery and excision of the right knee and right leg swellings was done under spinal anesthesia (Figure 5).

The postoperative period was uneventful, and the patient was discharged. Surgical site healed well and sutures were removed on postop day 7.

## Discussion

Subcutaneous mycoses refer to a heterogeneous group of diseases caused by a wide variety of fungi that invade the cutaneous and subcutaneous tissue after traumatic implantation and are common in tropical and subtropical countries like India. It comprises of zygomycosis, PHM, hylohyphomycosis, mycetoma, chromoblastomycosis, sporotrichosis, lobomycosis, and rhinosporidiosis [8]. Manifestations of subcutaneous myocosis are varied and present as a cyst, ulcer, scaly lesion, verrucous growth, warty plaque, abscess, keratotic macerated lesions, and subcutaneous nodular lesions. Of the different types of subcutaneous mycosis chromoblastomycosis, PHM and eumecotic mycetoma comprise dematiaceous fungi that are characterized by their dark pigmentation due to deposition of melanin in their cell wall [9].

Phaeohyphomycosis, a relatively new term introduced by Ajello in 1974, is caused by 109 species of fungi which are classified into 60 genera, of which commonly associated include Exophiala, Phialophora, Cladosporium, Curvularia, Fonsecaea, and Alternaria [2,10]. PHM can be classified into five types: (i) superficial, black piedra and tinea nigra; (ii) cutaneous: dermatomycosis and onychomycosis; (iii) mycotic keratitis; (iv) subcutaneous; and (v) invasive, systemic, and cerebral [6,11,12]. Of these, subcutaneous PHM is the most common type which clinically present as papulonodules, cysts, or abscesses [9]. It clinically presents as a painless single subcutaneous cyst and rarely as multiple cysts; the skin surface is smooth and uninvolved. These lesions average about 2.5 cm in size and commonly noted in the extremities [8]. PHM must be differentiated from other conditions such as chromoblastomycosis, mycetoma, ganglion cyst, epidermal inclusion cyst, baker’s cyst, foreign body granuloma, erythema nodosum, lipoma, and neurofibromas; this may be done with the assessment of clinical presentation followed by histopathology and culture sensitivity [9]. Histopathology of PHM shows inflammatory cells along with brown pigmented septate hyphae with acute angle branching. Hyphae measures 2-6 µm wide and they constrict at their prominent septations. Fontana-Masson staining demonstrates the presence of melanin; Gomori-Methenamine Silver and PAS stains also help in identifying the fungal wall [8,10]. However, the antifungal agents, including flucytosine and itraconazole, appear to be effective treatments for patients with subcuta­neous PHM [13]. Surgical excision of subcutaneous PHM is a widely accepted management for curative intent [6].

## Conclusions

Subcutaneous mycosis is common in tropical and subtropical countries like India. Strong suspicion of this diagnosis is warranted especially in immunocompromised patients. Histopathological study is the only way to diagnose a suspected case. The characteristics of the fungi can be identified using different types of stains. Antifungals can be used to alleviate the symptoms but rarely it cures the disease. Surgical excision is the treatment of choice to achieve early cure.
